# Re-Challenge with Ovalbumin Failed to Induce Bronchial Asthma in Mice with Eosinophilic Bronchitis

**DOI:** 10.1371/journal.pone.0075195

**Published:** 2013-09-20

**Authors:** Liyan Chen, Nanshan Zhong, Kefang Lai

**Affiliations:** 1 Department of Respiratory Medicine, the 1^st^ Affiliated Hospital of Guangzhou Medical University, Guangzhou, Guangdong, China; 2 Guangzhou Institute of Respiratory Disease, Guangzhou, Guangdong, China; 3 State Key Laboratory of Respiratory Disease, Guangzhou Medical University, Guangzhou, Guangdong, China; Murdoch University, Australia

## Abstract

**Objective:**

To investigate whether eosinophilic bronchitis without airway hyperresponsiveness will develop bronchial asthma in allergic mice.

**Methods:**

Mice were sensitized with OVA on days 0, 7, and 14, challenged on days 21 to 23 (1^st^ OVA challenge), and re-challenged on days 46 to 48 (2^nd^ OVA challenge), intranasally with 10 (the EB group) and 200 (the AS group) μg OVA. Lung resistance (R_L_) was assessed 24 h after each challenge and on day 45 followed by analysis of leukocyte distribution in the bronchoalveolar lavage (BAL) fluid and histological examination.

**Results:**

Twenty-four hours after the 1^st^ OVA challenge, aerosolized methacholine caused a dose-dependent increase in R_L_ in all groups. At doses ≥1.56 mg/mL, R_L_ in the AS group was significantly higher than that of the NS-1 group (*P*<0.01 or 0.05) and at doses ≥12.5 mg/mL, R_L_ was markedly higher in the AS group than that of the EB group (*P*<0.01). The percentage of eosinophils in both the EB group and the AS group was markedly higher than that of the control group. Twenty-four hours after the 2^nd^ OVA challenge, at doses ≤12.5 mg/mL, there was no significant difference in R_L_ among all groups (*P*>0.05). At doses ≥12.5 mg/mL, R_L_ in the AS group was significantly higher than that of the control group and EB group (*P*<0.01 or 0.05). The percentage of eosinophils in the AS group was noticeably higher than that of the EB group(*P*<0.05). Furthermore, there was apparent infiltration by inflammatory cells, predominantly eosinophils, into the sub-epithelial region of the bronchus and the bronchioles and around the vessels in the EB and AS group.

**Conclusion:**

Re-challenge with low doses of ovalbumin did not increase airway reactivity and failed to induce bronchial asthma in mice with ovalbumin-induced EB.

## Introduction

In 1989, Gibson et al. first described a particular group of patients who had chronic irritating dry coughs with scant sputum with increased numbers of eosinophils in induced sputum. These patients had normal ventilatory function with no airway hyperresponsiveness and had normal peak flow velocity rate. These patients responded well to glucocorticoid therapy. However, they did not fit the diagnostic criteria for bronchial asthma and the authors termed the condition eosinophilic bronchitis [1].

Even though the concept of eosinophilic bronchitis has been put forward for more than 20 years, it remains controversial whether eosinophilic bronchitis represents an independent disease entity or an early manifestation of asthma and whether eosinophilic bronchitis will develop into asthma. Berry*et al*. followed up 32 cases of eosinophilic bronchitis over 7 years and found that most of these patients had a protracted course [2]. Three cases (9%) developed asthma and 5 (16%) cases developed irreversible airway obstruction, suggesting that eosinophilic bronchitis may develop into asthma [2], but a very low rate of progression from eosinophilic bronchitis to an asthmatic phenotype. Park et al. followed up 24 cases of eosinophilic bronchitis who were treated with inhalational glucocorticoids and they found that only one patient developed asthma over the two-year follow up [3]. Given the growing prevalence of asthma globally, even a low rate of progression from eosinophilic bronchitis to asthma carries significant health implications and it is important to address whether this indeed occurs and the possible underlying mechanisms.

Asthma has been routinely described as an allergic disease over the decades in which it is believed that allergen exposure produces sensitization to allergens, and continued exposure leads toclinical asthma through the developmentof airways inflammation, reversible airflow obstruction, and enhanced bronchial reactivity. However, not all asthma cases are allergic and many cases may involve non-allergic mechanisms, including non-allergicinflammation of the airways. However, these non-allergic mechanisms are currently not well understood. The studies by Berry et al. and Park et al. [2,3]mostly focus on clinical manifestations of eosinophilic bronchitis and there have been few studies on the pathogenesis of the disease due to a lack of eosinophilic bronchitis animal models.

We were the first group to have established a mouse model of eosinophilic bronchitis which does not have airway hyperresponsiveness and exhibits characteristics of airway eosinophilic inflammation [4]. The model allows further studies of the pathogenesis of eosinophilic bronchitis and its relation with asthma. In the current study, we investigated whether mice with ovalbumin (OVA)-induced eosinophilic bronchitis developed into asthma and further characterized the relation between eosinophilic bronchitis and asthma by using mouse models of OVA-induced eosinophilic bronchitis and asthma.

## Materials and Methods

### Animals

Ninety 6-week old SPF-grade female BALB/c mice, weighing 16-18 g each, were provided by the Laboratory Animal Center of Guangdong Province, Guangzhou, China and were housed in SPF and environmentally controlled conditions (22°C, a 12 h light/dark cycle with the light cycle from 6: 00 to 18: 00 and the dark cycle from 18: 00 to 6: 00) at the National Key Laboratory of Respiratory Disease, Guangzhou Medical University, Guangzhou, China. The animals had *ad libitum* access to standard OVA-free laboratory chow and distilled water. The mice were housed for at least 1 week before investigation. This study was carried out in strict accordance with the recommendations in the Guide for the Care and Use of Laboratory Animals ofthe State Key Laboratory of Respiratory Disease. All experimental procedures were approved by the Animal Ethics Committee, The First Affiliated Hospital, Guangzhou Medical University (Approval ID: 00021647).

### Sensitization and intranasal challenge

**Figure 1:**
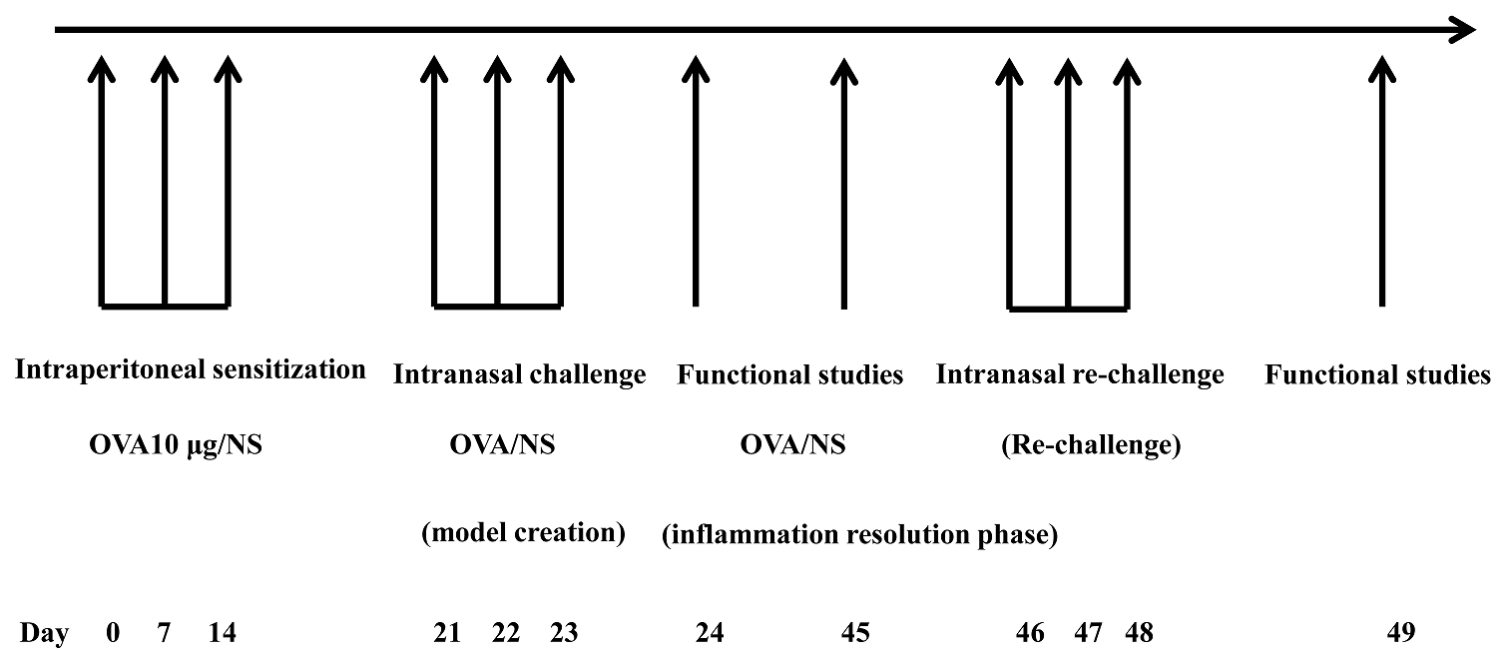
The study flow chart.

Mice were sensitized with chicken OVA (grade V) as previously described [4]. On days 0, 7, and 14, mice received an intraperitoneal injection of 10 µg OVA emulsified with 1.3 mg aluminum hydroxide (Sigma, St. Louis, MO) diluted with normal saline to a total volume of 200 µL. On days 21 to 23, they were anesthetized intraperitoneally with 1% pentobarbital sodium (90 mg/kg) and given an intranasal challenge with 10 (the EB-1 group) or 200 µg aerosolized OVA (the AS-1 group) in 50 µL with 25 µL in each nostril for 3 consecutive days. The control mice (the NS-1 group) received an equivalent volume of normal saline for sensitization and intranasal challenge. On days 46 to 48, mice in the EB group and AS group received a second intranasal challenge of 10 and 200 µg OVA, respectively, in 50 µL for 3 consecutive days while the control mice received an equivalent dose of normal saline. Assessment [5] of airway reactivity to methacholine (Sigma) followed by analysis of bronchoalveolar lavage (BAL) fluid and histological examination was performed on day 24 (the EB-1, AS-1 and NS-1 group), on day 45 (the EB-2, AS-2 and NS-2 group), and on day 49 (the EB-3, AS-3 and NS-3 group). The study protocol flow chart is shown in Figure 1.

### Determinations of airway reactivity

Airway reactivity was determined using the FinePointe RC System (Buxco). Mice were anesthetized with 1% pentobarbital sodium (90 mg/kg) and cannulated with an 18G blunted needle. Mice were placed in a body chamber and mechanically ventilated at a frequency of 120/min and a tidal volume of 0.2 mL. Baseline lung resistance (R_L_) was recorded for 3 min and expressed as mean RL = cm H_2_O/mL sec and changes in R_L_ were recorded for 3 min after challenge with aerosolized methacholine for 20 sec in 10-µL at a serial 2-fold increment from 0.39 to 50 mg/mL.

### Leukocyte distribution in the BAL fluid

Twenty-four hours after the last aerosol challenge with OVA or normal saline, the tracheae were cannulated and the lungs were lavaged three times with 0.8 mL PBS. The BAL fluid from each mouse was pooled and centrifuged at 1500 rpm for 10 min. The cell-free supernatant was stored at -80°C until further analysis and the pellet was resuspended and prepared for smears, which were fixed in 10% formalin and H&E stained. Cell types were identified by light microscopy with standard morphological criteria. Differential cell counts of 200 leukocytes were performed in triplicate.

### Light microscopy

After completion of BAL sampling, the lungs were inflated through the trachea with 0.8 mL10% formalin for at least 24 h and embedded in paraffinby immersion and were sectioned. The sections were deparaffinized in xylene and dehydrated in gradient ethanol (100% for 5 min [twice], 95% for 5 min, 85% for 5 min, and 75% for 5 min) and hematoxylin and eosin (H&E) stained. Tissue sections were examined under a light microscope by choosing 5 random fields at a magnification of 20 ×.

### Statistical analysis

Data were expressed as $\bar{x}\pm s$ and analyzed using the SPSS12.0 statistical software (SPSS Inc., Chicago, IL). Student’s *t* test was used for comparison of baseline R_L_ between two groups and one-way analysis of variance (ANOVA) was used for comparison of changes in R_L_ as well as changes in cell types in the BALF among multiple groups. LSD was employed for homogeneity of variance and Tamhane’s T2 test for heterogeneity of variance. Statistical significance was set at *P*<0.05.

## Results

### Ovalbumin-induced mouse asthma model exhibits enhanced lung resistance

**Figure 2:**
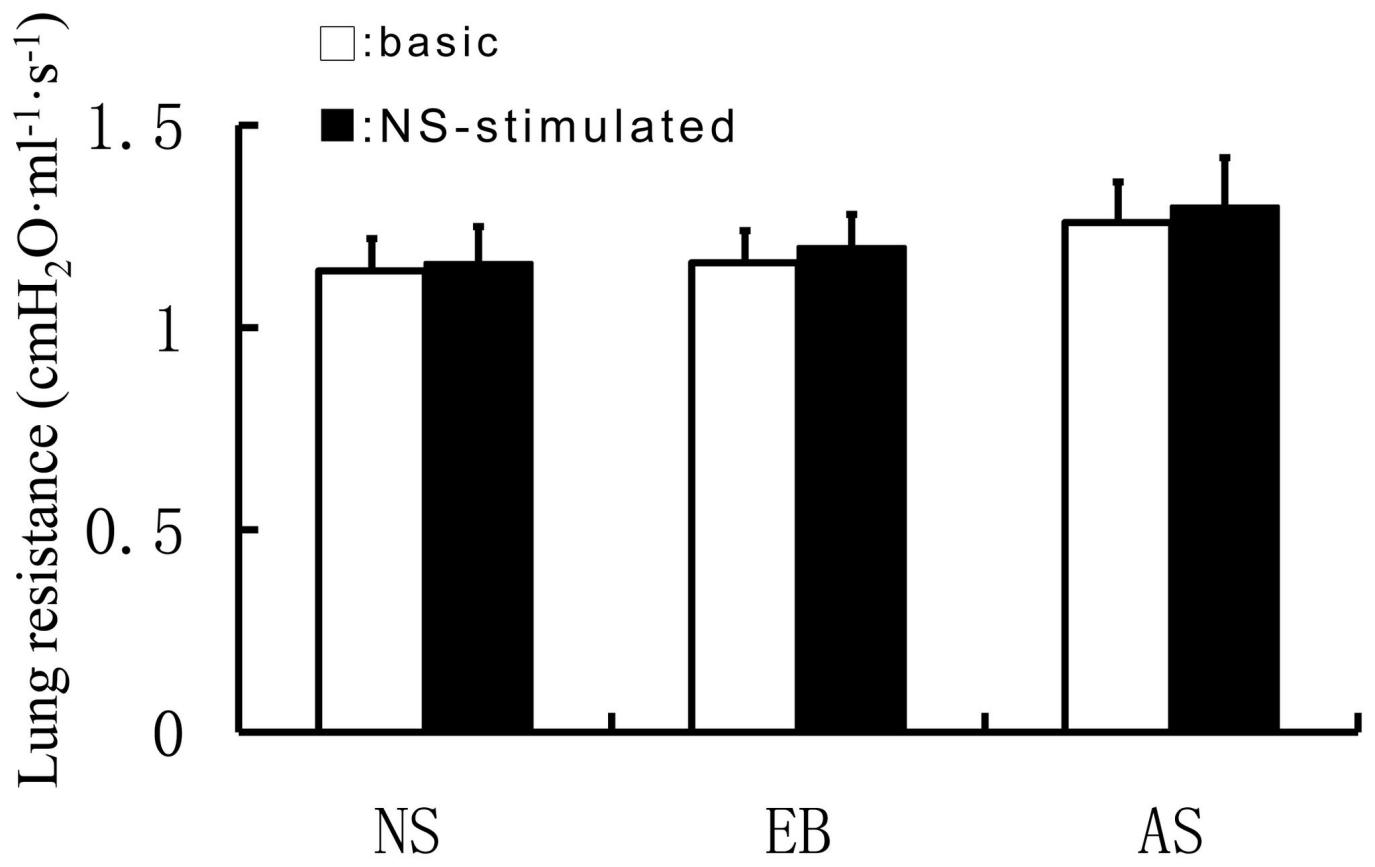
Comparison of lung resistance (R_L_) in mice when the models were set up and the normal saline (NS) control mice).

**Figure 3:**
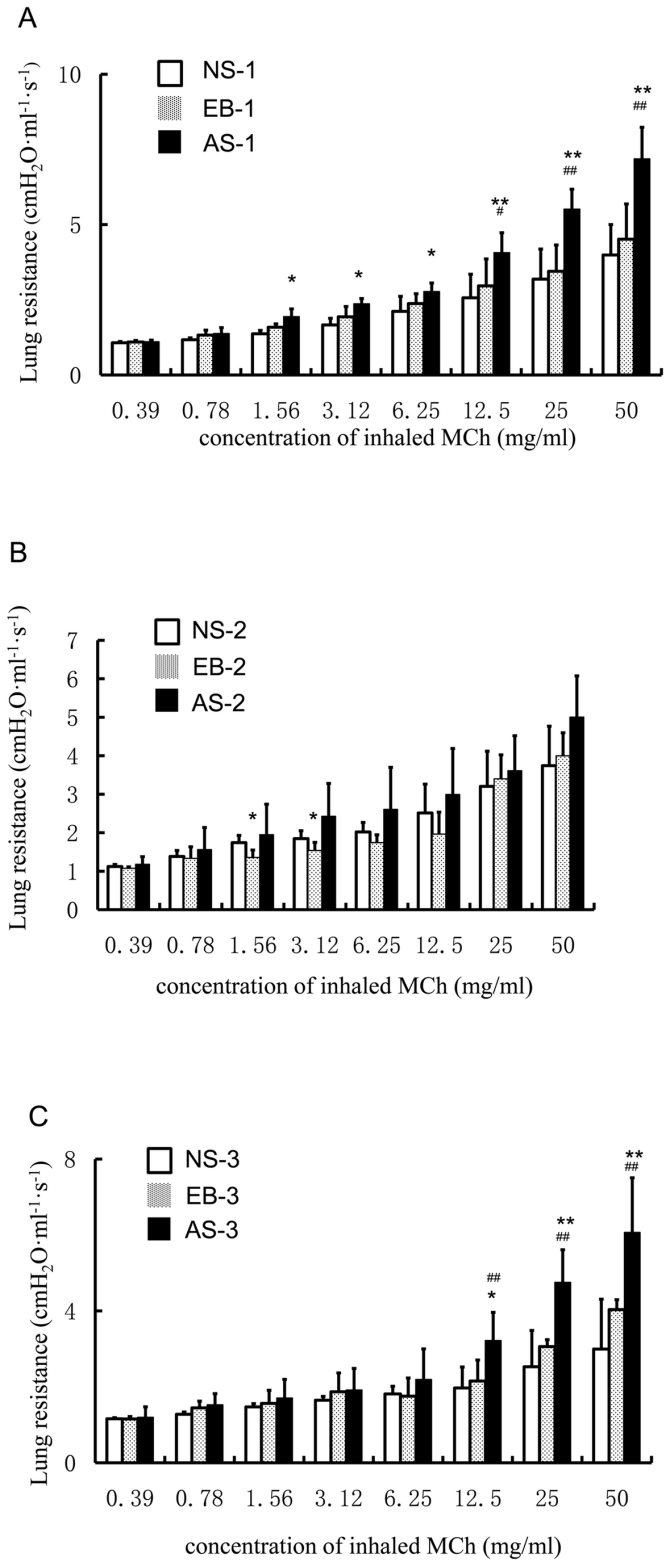
Changes in airway responsivenessin response to challenge with aerosolized methacholine. (A) Changes in lung resistance (R_L_) were recorded for 3 min after challenge with aerosolized methacholine on day 24, 24 h after the 1^st^ intranasal OVA challenge, for 20 sec at the indicated doses. Mice received 3 intraperitoneal injections of 10 µg of ovalbumin (OVA) emulsified with 1.3 mg of aluminum hydroxide on days 0, 7, and 14. They were then intranasally challenged with 10 (the EB-1 group) or 200 µg aerosolized OVA (the AS-1 group) for 3 consecutive days on days 21 to 23. The control mice received an equivalent volume of normal saline for sensitization and intranasal challenge. *P<0.05 and ** *P*<0.01, the AS-1 group vs. the NS-1 group; ^#^ *P*<0.05 and ^# #^ *P*<0.01, the AS-1 group vs. the EB-1 group. (B) Changes in airway reactivity in response to a second challenge with aerosolized methacholine. Changes in R_L_ were recorded for 3 min after challenge with aerosolized methacholine on day 45, 3 weeks after the first intranasal challenge, for 20 sec at the indicated doses. *P<0.05, the EB-2 group vs. the NS-2 group. (C) Changes in airway reactivity in response to challenge with aerosolized methacholine following the 2^nd^ intranasal OVA challenge. Changes in lung resistance (R_L_) were recorded for 3 min after challenge with aerosolized methacholine on day 49, 24 h after the 2^nd^ intranasal OVA challenge, for 20 sec at the indicated doses. *P<0.05 and ** *P*<0.01, the AS-3 group vs. the NS-3 group; ^#^ *P*<0.01, the AS-3 group vs. the EB-3 group.

We measured baseline R_L_ of mice in each group and found no marked difference in baseline R_L_ of mice amongthe NS group, the EB group and the AS group (data not shown). Upon challenge with normal saline, there was no more than 5% increase in R_L_ in all groups and the increase was comparable among all groups (Figure 2). We then challenged the mice with incremental doses of aerosolized methacholine on day 24, 24 h after the first intranasal OVA challenge. We found that aerosolized methacholine caused a dose-dependent increase in R_L_ in all groups (Figure 3A). At low doses of methacholine (0.39 and 0.78 mg/mL), no statistical difference was noted in R_L_ among the three groups. At doses of methacholine ≥1.56 mg/mL, R_L_ in the AS-1 group was significantly higher than that of the NS-1 group (*P*<0.01 or 0.05). At all doses of methacholine, there was no statistical difference in R_L_ between the EB-1 group and the NS-1 group. By contrast, at doses of methacholine≥12.5 mg/mL, R_L_ was markedly higher in the AS-1 group than that of the EB-1 group (*P*<0.01). These findings indicate that mice with OVA-induced asthma exhibited increased lung resistance.

On day 45, 3 weeks after the first intranasal OVA challenge, we examined the lung function of mice presented as RL to determine whether the lung function had recovered from the intranasal challenge. We found that aerosolized methacholine caused a dose-dependent increase in R_L_ in both the EB-2 and AS-2 group (Figure 3B). At low (0.39 to 1.56 mg/mL) and high (25 and 50 mg/mL) doses of methacholine, no statistical difference was noted in R_L_amongtheNS-2, EB-2 and AS-2 group. At doses of methacholine between 3.12 and 12.5 mg/mL, R_L_ in the EB-2 group was significantly lower than that of the NS-2 group (*P*<0.01). Of note, mice in both EB-2 and AS-2 group showed decreased R_L_ compared with that of mice in the EB-1 and AS-1 group on day 24. Furthermore, at each dose of aerosolized methacholine, R_L_ in the AS-2 group showed no statistical difference from that of the NS-1 group and EB-1 group on day 24.

We then carried out a second challenge with aerosolized methacholine on day 46, 47 and 48.We further challenged the mice with incremental doses of aerosolized methacholine on day 49, 24 h after the second intranasal OVA challenge. We found that aerosolized methacholine caused a dose-dependent increase in R_L_ in all groups (Figure 3C). At doses of methacholine ≤12.5 mg/mL, there was no significant difference in R_L_ among all groups (*P*>0.05). At doses of methacholine ≥12.5 mg/mL, R_L_ in the AS-3 group was significantly higher than that of the NS-3 group and EB-3 group (*P*<0.01 or 0.05).

### Mice with OVA-induced asthma showed increase in the percentage of eosinophils in the BAL fluid

**Figure 4:**
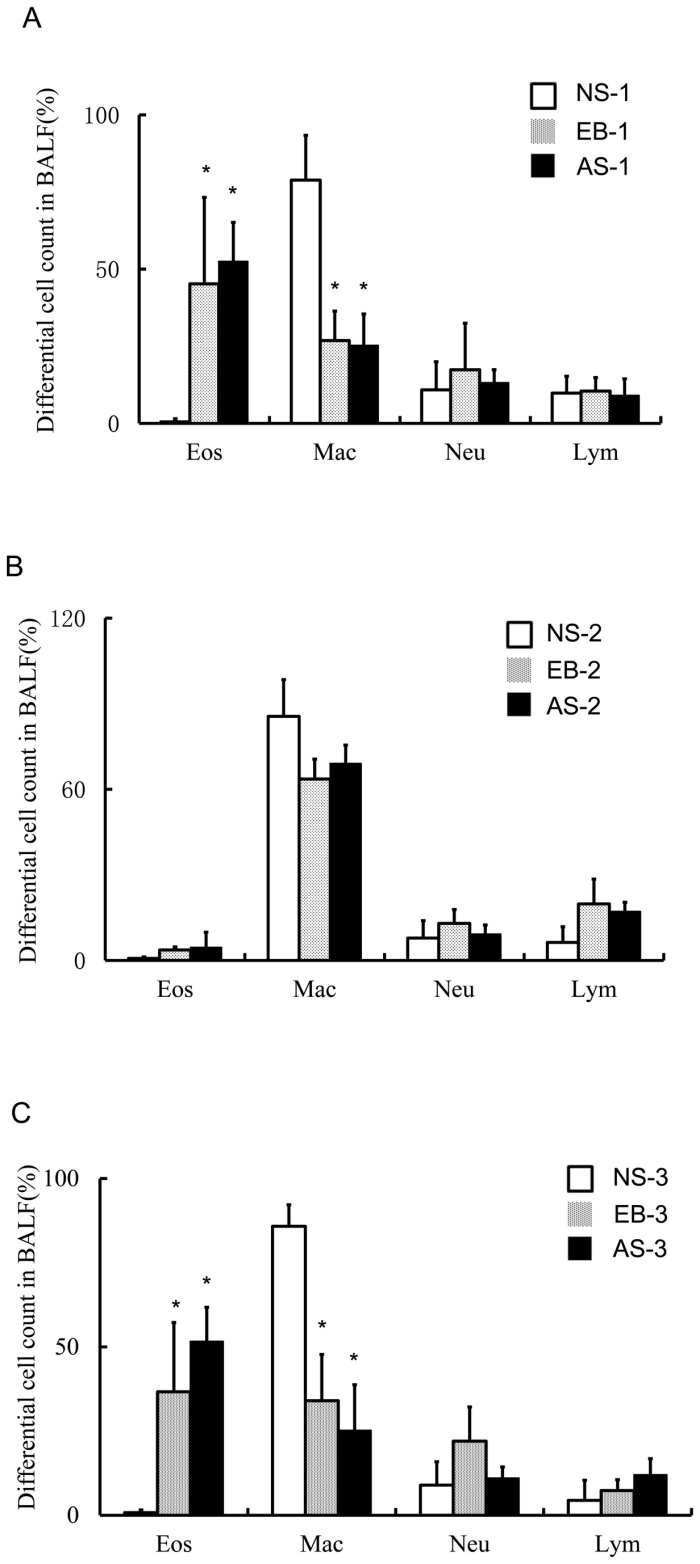
Leukocyte distribution in the bronchoalveolar lavage (BAL) fluid. (A) Differential cell counts of 200 leukocytes were performed in triplicate for smears prepared of cells in the BAL fluid on day 24, 24 h after the 1^st^ intranasal OVA challenge. * *P*<0.01 vs. the NS-1 group, and (B) on day 45, 3 weeks after the first intranasal OVA challenge, and (C) on day 49, 24 h after the 2^nd^ intranasal OVA challenge. * *P*<0.01 vs. the NS-3 group.

We determined leukocyte distribution in the BAL fluid collected on day 24, 24 h after the 1^st^ intranasal OVA challenge. The percentage of eosinophils in both the EB-1 group and the AS-1 group was markedly higher than that of the NS-1 group (Figure 4A) (*P*<0.05). By contrast, the percentage of macrophages was significantly lower in both the EB-1 group and the AS-1 group than that of the NS-1 group (*P*<0.05) while no apparent differences were observed in the percentages of neutrophils and lymphocytes among all three groups (*P*>0.05). We then determined leukocyte distribution in the BAL fluid collected on day 45, 3 weeks after the first intranasal OVA challenge. The percentage of eosinophils was markedly reduced compared with that on day 24 in both the EB-2 group and the AS-2 group (Figure 4B). There was no statistical difference in the percentages of macrophages, lymphocytes and neutrophils between the AS-2 group and group EB-2 group. We further determined leukocyte distribution in the BAL fluid collected on day 49, 24 h after the second intranasal OVA challenge. We found no statistical difference in the percentages of eosinophils, macrophages, lymphocytes and neutrophils between the AS-3 group and group EB-3 group (*P*>0.05). Furthermore, upon re-challenge, the percentage of eosinophils in the BALF rose again in the EB-3 and AS-3 group, which showed no significantly statistical difference from that when the model was established (Figure 4C).

### Mice with OVA-induced asthma showed increased infiltration of eosinophils in the lung tissues

**Figure 5:**
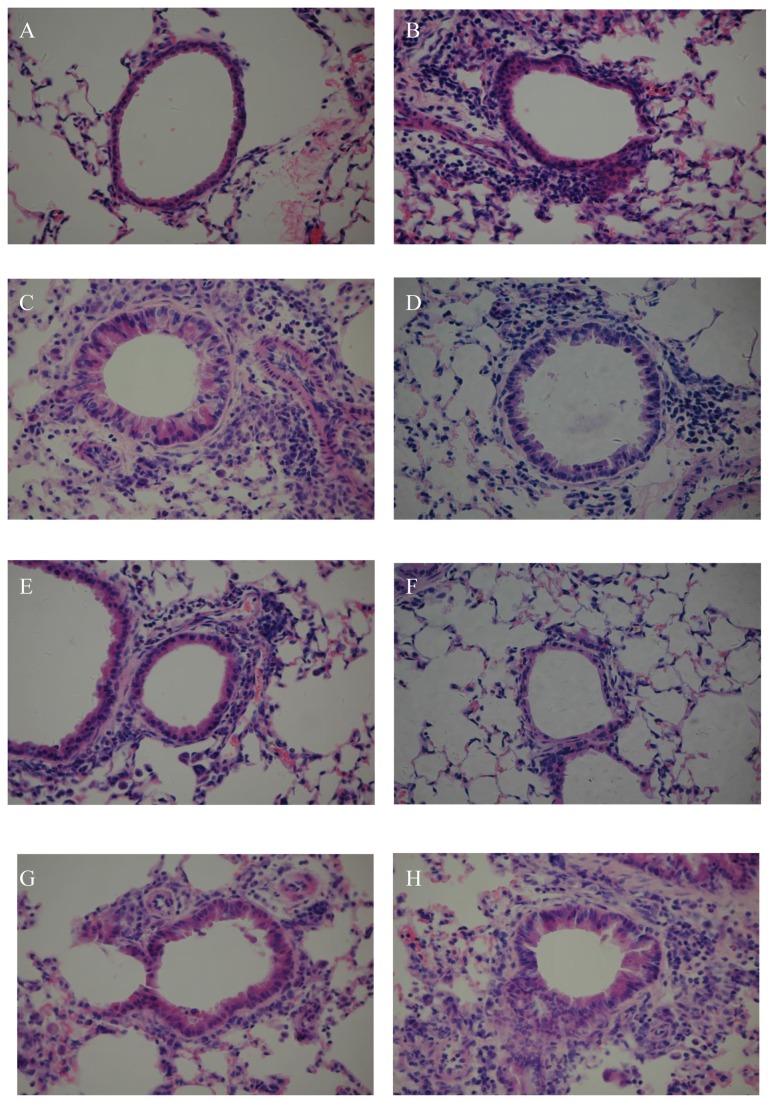
Pathologic changes in the lung tissues fromOVA-challenged mice. (A) NS-1; (B) EB-1; (C) AS-1; (D) EB-2; (E) AS-2; (F) NS-3; (G) EB-3; (H) AS-3. Mice in the EB groups and AS groups showed engagement of blood vessels, infiltration by eosinophils. H and E, ×200.

In the NS groups, the lung structure was clear and there was no infiltration by inflammatory cells of the alveolar wall and the bronchial wall and there was no perivascular infiltration. Furthermore, there was no congestion in the interstitium. In the EB groups and AS groups, there was apparent infiltration by inflammatory cells, predominantly eosinophils, into the sub-epithelial region of the bronchus and the bronchioles and around the vessels. In addition, epithelial cells became detached and there was increased exudation into the alveolar cavity. Histiocytes or macrophages were observed and there was congestion of the interstitium (Figure 5).

## Discussion

In the current study, we sensitized mice by intraperitoneal injection of 10 µg OVA followed by intranasal challenge with 10 µg OVA to establish a mouse model of eosinophilic bronchitis. Mice of this model showed no apparent difference in R_L_ compared with the control mice. By contrast, compared with that of the controls, airway reactivity markedly increased in mice receiving an intranasal challenge of 200 µg OVA. In addition, mice in the EB and AS group showed a marked increase in the proportion of eosinophils in the BAL fluid and increased infiltration of inflammatory cells in the lung tissues, indicating that the animal models were successfully established. Three weeks after the animal models were established, R_L_ of mice in the EB group and the proportion of eosinophils in the BAL fluid were lower than those of mice on day 24 in response to 3.12-12.5 mg/mL methacholine. Furthermore, mice in the AS group showed no apparent difference in airway reactivity from the control mice, and the infiltration of eosinophils in the lung tissues also became less noticeable. These findings suggest that inflammation in the mice had largely subsided.

Jeroen*et al*. established a mouse model of asthma using tolylenediisocyanate and observed noticeably increased airway reactivity upon the first challenge; however, the amplitude of increase in airway reactivity declined with subsequent challenges, which they attributed to the development of immune tolerance as a result of multiple challenges [6]. Chung et al. found that prior oral administration of antigens suppressed airway hyperresponsiveness, the development of antigen-specific IgE, the production of TH_2_ cytokines, antigen-mediated T cell proliferation and infiltration of inflammatory cells [7]. Shi et al. studied leukocyte distribution in the BAL fluid using aerosolized OVA-challenged mice and they found that the number of inflammatory cells was markedly reduced by persistent or intermittent OVA challenges, which was related to the development of allergen-dependent immune tolerance. Natarajan et al. used LPS to induce long-lasting immune tolerance in mice, and they found lessened inflammation associated with asthma caused by cockroach allergens [8].

Current studies on immune tolerance of allergic diseases are mostly preventative in nature and there have been few studies on immune tolerance by re-challenge after asthma is established. In the current study, we established mouse models of eosinophilic bronchitis and asthma and re-challenged the mice with intranasal OVA. We observed no apparent difference in airway responsiveness between mice in the EB-3 group and EB-1 group and between mice in the AS-3 group and AS-1 group though airway responsiveness was subdued, suggesting that eosinophilic bronchitis failed to evolve into asthma against low doses of OVA while asthma was not aggravated by high doses of OVA. Chung et al. found that multiple high doses of toleragens suppressed the progression of mild asthma and reduced the number of eosinophils in the BALF and decreased airway responsiveness; however, these effects were not observed in severe asthma mouse model (6). Palmqvist*et al*. found that low doses of toleragens lessened delayed type hypersensitivity and lowered airway reactivity (11). Zhang et al. found that high doses of DNA vaccines suppressed airway reactivity, indicating that high doses of antigens could prevent TH2 biased immune response by inducing Tregs, thereby suppressing the development of asthma (12).

In the current study, no marked difference in the percentage of eosinophils in the BALF was observed at the time of model creation and OVA re-challenge of mice in the EB group and AS group, indicating that enhanced airway responsiveness in allergic asthma is not related to eosinophil levels. Therefore, both eosinophil levels and airway responsiveness should be examined in establishing asthma models. We re-challenged mice with 10 µg OVA for 3 consecutive days. Even though the proportion of eosinophils in the BAL fluid and the lung tissues in the EB-3 group increased, airway reactivity showed no apparent difference from that of the EB-1 group and was markedly lower than that of the AS-3 group, which were re-challenged with 200 µg OVA for 3 consecutive days, and the AS-1 group. These findings showed that persistent challenges with low doses of OVA failed to enhance the airway reactivity of mice in the EB group and failed to promote the development of eosinophilic bronchitis into asthma, suggesting that eosinophilic bronchitis is likely an independent disease entity. We speculate that this could be related to the possibility that multiple low-dose intranasal challenges have led to the development of immune tolerance in the mice. Van Hove et al. and Tournoy*et al*. found that prolonged OVA exposure led to a complete loss of airway inflammation, possibly related to inhibition of maturation of dendritic cells (DCs) and suppression of cross stimulation of DCs and T lymphocytes, which could lead to the exhaustion of the immune system [9,10]. Furthermore, we found no statistical difference in the percentage of eosinophils in the BAL fluid of mice in the EB group and AS group on days 24 and 49, implying that enhanced airway reactivity in asthma is not related to the number of eosinophils. Therefore, in animal models of asthma, eosinophils and airway reactivity should both be determined.

In conclusion, the mouse model of eosinophilic bronchitis can be established with low doses of OVA. Re-challenge with low doses of OVA after airway inflammation has resided failed to promote the development of eosinophilic bronchitis into asthma, indicating that eosinophilic bronchitis is an independent disease entity.
